# Fracture resistance and mode of failure of MOD cavities restored with short glass versus polyethylene fiber-reinforced composites: an in vitro study

**DOI:** 10.1186/s12903-026-07999-z

**Published:** 2026-03-25

**Authors:** Saeed Mosa Fadah, Rehab Khalil Safy, Basma Hosny Mohamed

**Affiliations:** Conservative Dentistry Department, Faculty of Dentistry, Suez Canal University, Ismailia, Egypt

**Keywords:** Dental restoration, Resin composite, Short fiber-reinforced composite (SFRC), Polyethylene fibers, Tooth fracture, In vitro techniques

## Abstract

**Background:**

To improve the strength of large cavities, different types of fiber reinforcements are used. Polyethylene and glass fibers are two popular options, each with its own set of benefits. This study evaluated the fracture resistance and failure mode of two types of fiber reinforcements: short glass fiber-reinforced composites and ultra-high molecular weight polyethylene fibers in MOD cavities.

**Methods:**

To evaluate fracture resistance, eighty sound extracted lower third molar teeth were randomly divided into five groups (*n* = 16): intact teeth (positive control) (R1), unrestored teeth (negative control group) (R2), nanohybrid resin composite group (R3), SFRC group (R4), and Ribbond fibers group (R5). Teeth in groups R2, R3, R4, and R5 received standardized MOD cavities and were restored accordingly. R2 group left unrestored. In group R3, the prepared cavities were restored using a nanohybrid resin composite, in group R4, flowable SFRC was used as a base with nanohybrid composite layered over it. In group R5, a piece of Ribbond meshwork was applied in a U-shaped pattern, followed by the application of nanohybrid composite. Fracture resistance was determined using a universal testing machine. Following fracture, the teeth were examined visually and photographed via camera to determine the mode of failure.

**Results:**

Intact teeth had the highest fracture resistance (3201.4 ± 123.4), followed by the SFRC group, the Ribbond fiber group, and the nano-hybrid resin composite group with values of 2132.9 ± 169.5, 2084.5 ± 177.2, and 1576.8 ± 118.3, respectively. Unrestored teeth had the lowest mean fracture resistance value of 354 ± 32.8. There was a statistically significant difference among all groups (*P* < 0.001), except for SFRC group and Ribbond fibers group which did not differ significantly (*p* = 0.436). Regarding failure mode results, intact teeth showed the highest repairability rate (75%), followed by the SFRC group (62.5%), the Ribbond fibers group (50%), and the nanohybrid resin composite group (31.3%). The unrestored teeth had the lowest repairability rate (12.5%). In terms of intergroup comparison, there was a significant difference among all groups (*p* = 0.05).

**Conclusions:**

Short glass and polyethylene fiber-reinforced composites have a positive impact on the fracture resistance and repairability of restored MOD cavities.

## Background

Posterior teeth are subjected to various factors that affect their fracture resistance, such as long-standing caries, trauma, noncarious lesions, extensive cavity preparation, and root canal therapy [1]. Class II cavity preparations have proven to be challenging, especially when both proximal surfaces are involved [2], such as in the case of lost marginal ridges. MOD preparations diminish the fracture resistance of teeth by 54% compared to sound teeth. Furthermore, deep MOD cavities had significantly lower fracture resistance, regardless of the thickness of the remaining walls.[3].

The durability of restorative materials is influenced by their resistance to failure, which is evaluated through fracture resistance testing. Fracture resistance refers to the ability of a restored tooth to withstand functional occlusal forces and resist fracture under loading conditions. Therefore, dental practitioners are focused on improving the mechanical properties of restorative materials such as fracture toughness, flexural strength, and bonding to tooth structure to enhance the overall performance of restorations in structurally compromised teeth. According to the literature, resin composites have the potential to restore fracture resistance and strengthen teeth by acting as internal splints in small to moderate cavities [4]. However, in wide and deep cavities, this is questionable due to insufficient toughness, wear, and polymerization shrinkage of the resin composite material, which results in marginal gapping, staining, microleakage, and recurrent caries [5]. 

Several methods were used to overcome the previous shortcomings of conventional resin composites, to be used in wide and deep cavities. One of these methods was fiber incorporation, which extends their possible applications by internally strengthening restorations and reducing crack initiation [6]. Polyethylene ribbon and glass fibers are the most commonly used types of fiber-reinforced composites (FRCs) and are crucial for enhancing the fracture resistance of restored teeth [7]. An ultra-high molecular weight polyethylene (UHMWP) fiber is treated with cold gas plasma to strengthen its chemical bond to the resin composite materials and to act as a layer that absorbs the stresses internally and splints the restored teeth [7]. 

Additionally, glass FRCs have been shown to improve fracture resistance and limit crack propagation in resin composite restorations. SFRC have been shown to improve fracture resistance when used as a base material in high stress bearing restorations [8]. Multiple studies have investigated the reinforcing effect of different fiber systems on the mechanical behavior of direct composite restorations. Most of these reports demonstrated that fiber reinforcement enhances fracture resistance and shifts failure patterns toward more repairable fractures [9]. However, some investigations found no significant improvement, suggesting that the reinforcing effect may depend on fiber type, configuration, and placement technique [10]. Short randomly oriented glass fibers (SFRC) act as internal crack stoppers, limiting crack propagation within the restoration and improving load distribution, whereas long or continuous polyethylene fibers (Ribbond) primarily function by splinting the cusps and redistributing tensile and shear stresses along the axial walls. In addition, fiber incorporation has been shown to mitigate polymerization shrinkage stresses, which contribute to crack initiation at cavity margins. These combined effects influence the likelihood that a fracture will remain above the cemento-enamel junction (CEJ), leading to a more favorable or “safe-zone” failure pattern that is clinically repairable rather than catastrophic [11-13]. Therefore, the current study aimed to evaluate the fracture resistance and mode of failure of MOD cavities restored with short glass vs. polyethylene fiber-reinforced composites. The null hypothesis of the current study was that there was no significant difference between the fracture resistance and the mode of failure of teeth restored with nanohybrid resin composites reinforced with UHMWPs, as well as those restored with short glass fiber flowable and nanohybrid composites.

## Materials and Methods

### Materials

Materials used in the study were listed in Table 1.

**Table 1:** Materials used in the study

Materials	Description	Compositions	Manufacturers	Batch numbers
N-Etch	Etching gel	37% phosphoric acid	(Ivoclar Vivadent, Asia)	Z041SK
Tetric N-bond universal	Universal bonding agent	Bis GMA (25–50%), water and ethanol (10-<25%), 2-hydroxyethyl methacrylate (HEMA) (10-<25%), phosphonic acid methacrylate (MDP) (10-<25%), diphenyl (2,4,6-trimethylbenzoyl) phosphine oxide (1-<2.5%), urethane dimethacrylate.		Z041LS
Tetric N-ceram	Nano-hybrid resin composite	The resin matrix is composed of dimethacrylates (19–20 weight%) and fillers such as barium glass, ytterbium trifluoride, mixed oxide, and copolymers (80–81 weight%). Additives, initiators, stabilizers, and pigments make up less than 1 wt%. The total volume of inorganic fillers is 55–57%.		Z03Z58
everX Flow™	SFRC	The resin matrix consists of Bis-MEPP, TEGDMA, and UDMA, while the fillers are a combination of short E-glass fibers and particle fillers, primarily barium glass. fiber content ≈ 17 wt% and total filler load ≈ 70 wt% (including glass fibers and particulate fillers (.	(GC company, Tokyo, Japan)	2112071
Ribbond^®^ Ultra	Fiber meshwork	Ultrahigh molecular weight polyethylene fibers.	(Ribbond, Seattle, WA, USA)	D758U0
CERAMAGE Modelling liquid	Ribbond wetting resin	UDMA, 2-HEMA, Trimethylolpropane trimethacrylate.	(SHOFU, Kyoto, Japan)	092181

### Methods

#### Study design and sample size calculation

After being waved from the ethical committee at the Faculty of Dentistry Suez Canal University (no #273/2022), this in-vitro study was carried out on freshly extracted human-impacted third molar teeth. One-way ANOVA was used to assess differences among the five groups. A minimum total sample size of 80 samples was sufficient to detect the effect size of 0.40 according and a power (1-β = 0.80) of 80% at a significance probability level of *p* < 0.05 partial eta squared of 0.14. According to sample size calculations each treatment group was represented by a minimum of 16 samples.

#### Selection of teeth

Eighty sound permanent human-impacted lower third molar teeth extracted from patients aged 20–30 years for orthodontic reasons were collected according to the eligibility criteria for tooth selection where [14] all selected teeth were free of caries, cracks and dental anomalies. Any teeth showing resorption or open apices were excluded from the study and replaced.

Following extraction, teeth were cleaned thoroughly under running water to remove blood and mucus, and any remaining periodontal ligaments, and before cavity preparation, each tooth was gently polished using a low-speed rubber cup with non-fluoridated pumice paste to remove any debris or stains, ensuring a standardized and clean enamel surface without altering its morphology [15]. The teeth were examined for freedom of cracks via a magnifying lens X3.

To minimize the influence of size and shape variations on the results, teeth of almost the same size and shape were used. The buccolingual and mesiodistal widths were measured in millimeters at the most prominent points of the crown with a digital caliper (SHAN, Japan). To prevent the use of outliers, teeth below or above the average size boundaries (mean ± standard deviation (SD)) of 12.1 ± 0.63 mm mesiodistally and 10 ± 0.5 mm buccolingually were excluded. After that, the teeth were kept in buffered saline with 0.5% thymol for no more than a month [14]. 

#### Teeth mounting and preparation

A cylindrical Teflon mold with a diameter of 2 cm and a height of 2 cm was used for mounting teeth on acrylic resin blocks. To simulate the periodontium, the roots of all teeth were coated with melted wax (Cavex, Holland B.V) to a depth of 2 mm apical to the cementoenamel junction, creating a uniform 0.3 mm coat around the root. After the wax had hardened, each tooth was embedded in a self-curing acrylic resin cylinder block (Acrostone, Egypt). With the aid of a periodontal probe (UNC-15, Paterson Dental), the amount of acrylic resin used was adjusted to 2 mm below the cementoenamel junction (CEJ) of each tooth [16]. 

**Fig. 1:**
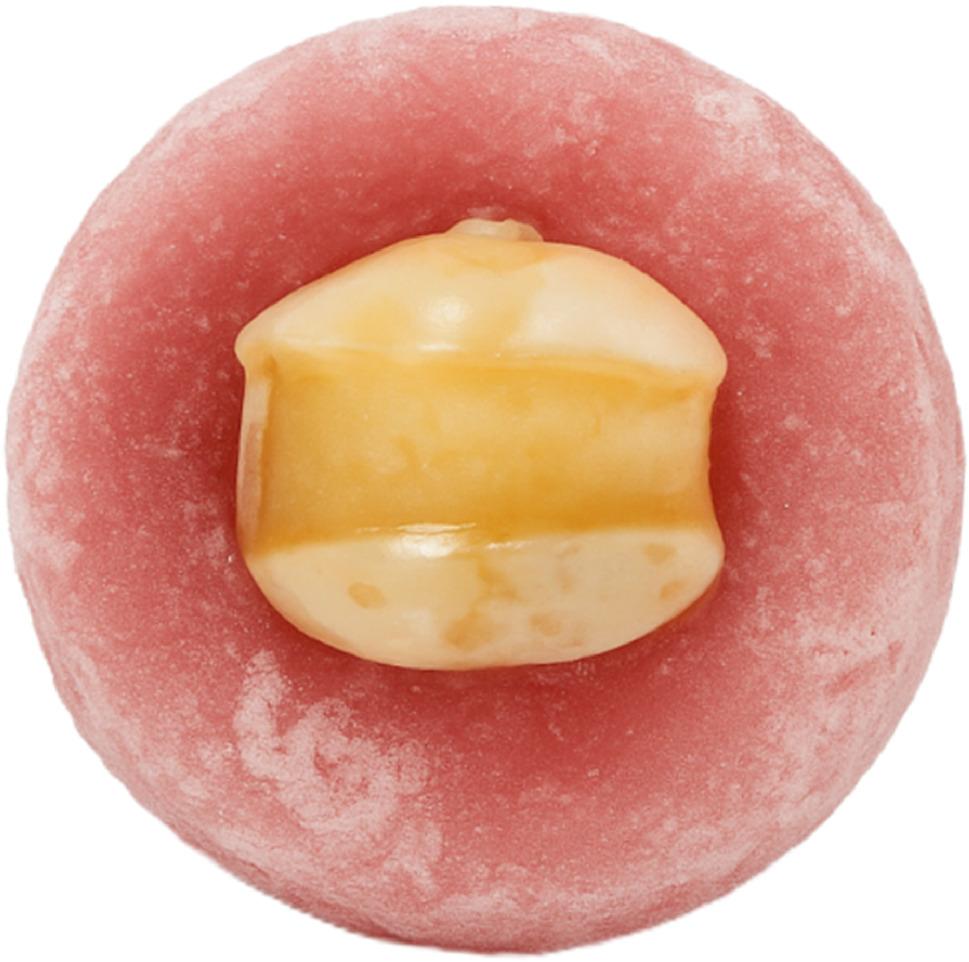
Cavity Design

After randomization, groups were coded as R1–R5, where ‘R’ denotes ‘Restoration group,’ followed by the group number representing the corresponding restorative condition. Sixteen teeth were kept intact to act as the positive control (R1). The remaining four groups received standardized mesio-occulso-distal cavities (MOD) using a diamond flat-ended fissure bur (Brasseler USA Dental, GA, USA) mounted in a high-speed handpiece with copious airwater spray. All cavities’ dimensions were standardized as follows: The cavity had a depth of 4 mm, a buccolingual width was 4 mm, and the buccal and lingual walls were 2 mm thick [15]. All internal line angles were rounded, the cavo-surface margin was prepared at a 90° angle, and the lingual and buccal walls were parallel to each other. The gingival floors located 1 mm above the CEJ. Mesial and distal boxes were identical in dimensions, and all floor levels were kept uniform [15]. Each bur was discarded after five preparations. A light air-water spray was utilized to clean all the cavities from the remaining debris, and a digital caliper was used to verify that each tooth was prepared with the same depth and width. (Figure 1) Following cavity preparation, the teeth were stored in buffered saline containing 0.5% thymol for a maximum of one month to prevent microbial growth; all samples were restored within 1–2 days after preparation [14]. 

#### Restoration of teeth

Immediately following preparation, 16 teeth were left unrestored to act as the negative control group (R2). A selective enamel etching technique was used in the remaining groups. Enamel margins were etched with 37%phosphoric acid gel for 30s, rinsed thoroughly, and gently air-dried, while no etching was applied to dentin. Each tooth was encircled by a circumferential metal matrix that had been securely adjusted (Automatrix^®^ MT, Dentsply, Milford, DE, USA), then a universal adhesive (N-Etch, Ivoclar Vivadent, Asia) was applied to both enamel and dentin and rubbed on the cavity floor and walls for 10s via a disposable brush. A gentle air stream was applied for 5s to ensure that the solvent was fully evaporated, and then the universal adhesive was polymerized via LED light-curing unit (Elipar S10, 3 M ESPE, St Paul, MN, USA) operating at a light intensity of 1200 mW/cm^2^ real irradiation output for 20s according to the manufacturer’s instruction. The light intensity of the LED curing unit was verified using a digital radiometer (Bluephase Meter II, Ivoclar Vivadent, Liechtenstein) before each curing session, and the average output was maintained at approximately 1200 mW/cm² [17]. 

Restoration of the nanohybrid resin composite group (R3) was performed through building up of the missing walls by incremental application of nanohybrid resin composite restorative material (Tetric N-Ceram, Ivoclar Vivadent, Asia); each increment was 2 mm thick and light cured for 20s according to the manufacturer’s instructions, after which the circumferential metal matrix was removed. Then, the proximal walls were post cured for 20s.

Then, the nanohybrid resin composite was applied incrementally (2 mm thickness) and adapted to the cavity walls using a ball and pear shape instrument (LM-Dental, Parainen, Finland). Each increment was light cured for 20s, where the light curing tip was maintained as close as possible to the restorative material.

**Fig. 2:**
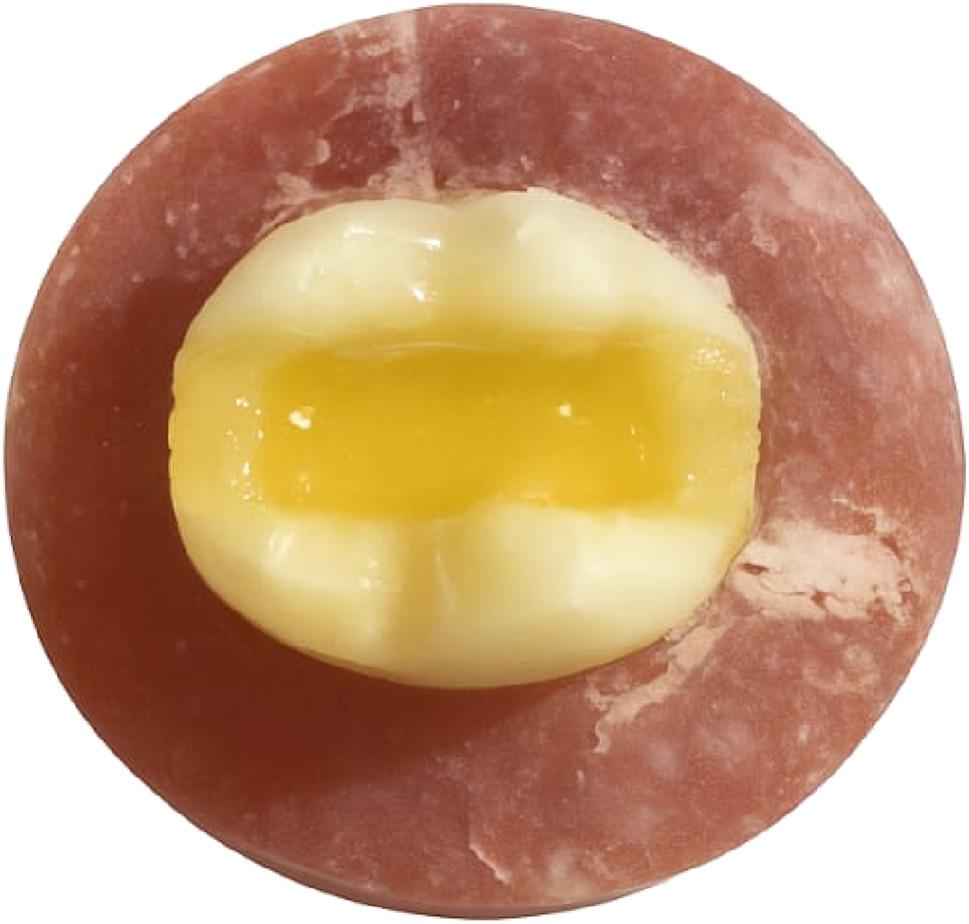
SFRC- base

**Fig. 3:**
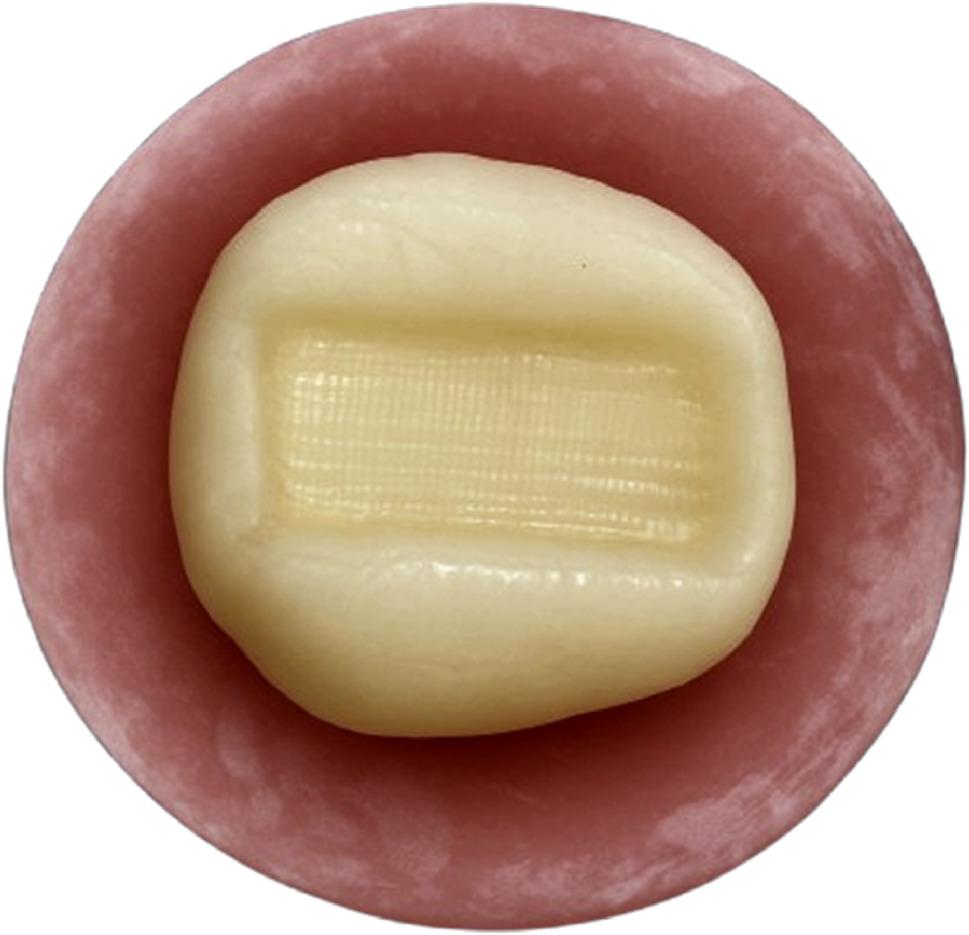
Ribbond-base

**Fig. 4:**
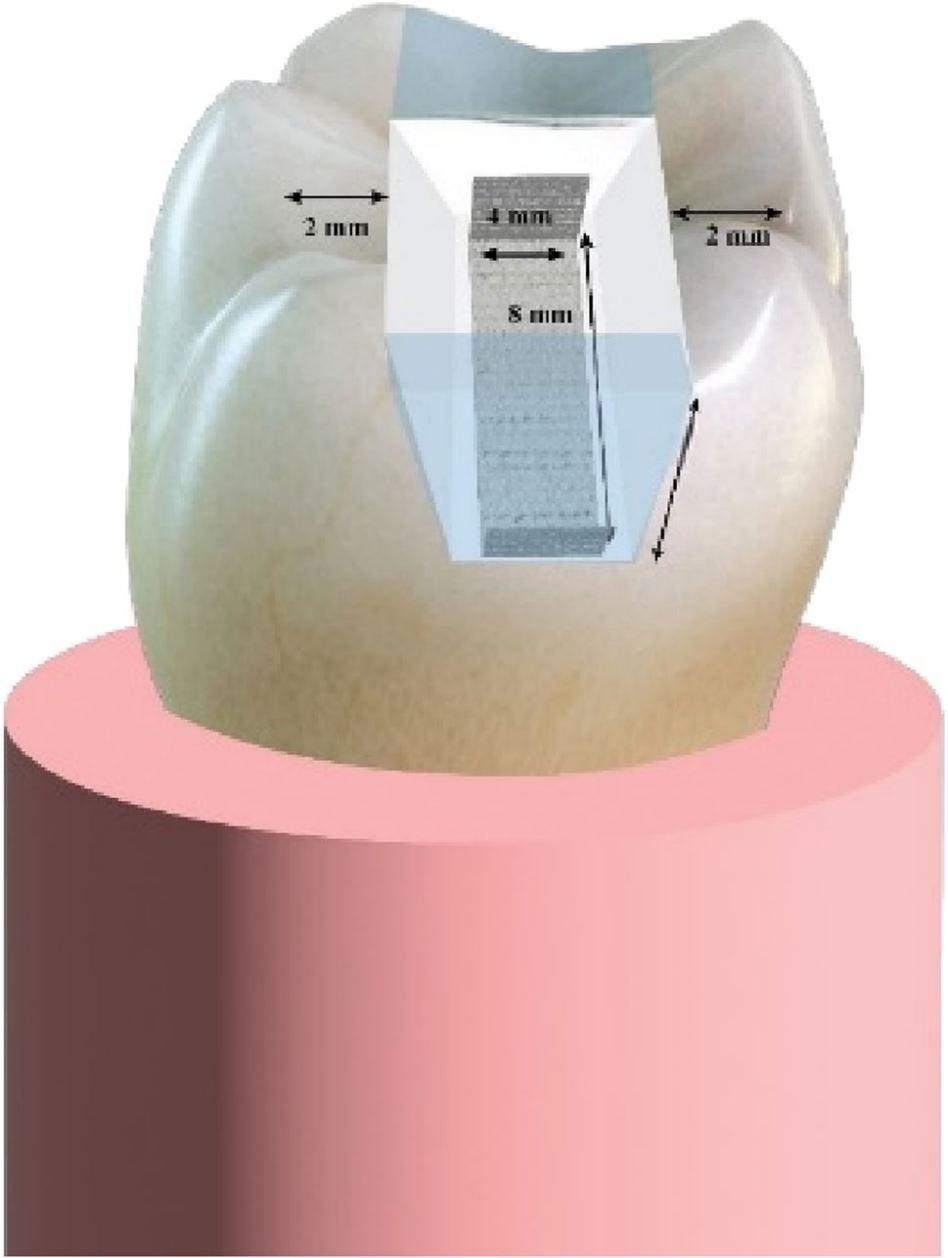
Schematic illustration of Ribbond application

Later on, restoration of the SFRC group (R4) was performed through the utilization of SFRC (everX flow, GC Europe, Leuven, Belgium) as a base. The flowable base material was applied in single increments of 2 mm thickness, leaving 2 mm occlusally for the final resin composite restoration, as recommended by the manufacturer. This base material was then light cured for 20s from the occlusal surface. Immediately after the base material was cured, the nanohybrid composite (Tetric N-Ceram) was applied incrementally as the final occlusal layer and light cured for 20s and the cavity was restored as mentioned in the R3 group. (Figure 2) Finally, restoration of Ribbond fibers group (R5) was performed through the application of a piece of UHMWP fibers (Ribbond, Seattle, WA, USA). The Ribbond length was 8 mm, and the width was 4 mm which was determined using periodontal probe. The predetermined piece of Ribbond fibers was cut, wetted with unfilled resin (CERAMAGE Modelling Liquid, SHOFU, Japan), and air-dried. Then the fiber meshwork was embedded in a layer of uncured nanohybrid resin composite that was applied on the floor and both proximal walls. The Ribbond was then placed against it to cover the entire pulpal floor and extend part way up to the mesial and distal surfaces in a U-shaped pattern. (Figures3 and 4) Then it was cured for 20s according to the manufacturer’s instructions, after which the cavities were restored as mentioned in the R3 group [18]. 

All restored groups had the same finishing and polishing procedures using a fine granular diamond bur (MANI, Utsunomiya, Japan) for finishing and ENA polishing paste (Micerium, Avegno, Italy) with rubber cups (KENDA, Liechtenstein) for polishing. After finishing and polishing, all the samples were stored in buffered saline with 0.5% thymol until they were tested for no more than a month [14]. 

#### Thermocycling procedures

All teeth were removed from the acrylic blocks before thermocycling and then were thermocycled in a water bath at 5 °C followed by 55° C for a total of 1000 cycles for a 20s dwell time each and an intermediary 5s resting time (SD Mechatronic thermocycler, Germany) [19]. 

#### Fracture resistance test and failure mode assessment

**Fig. 5:**
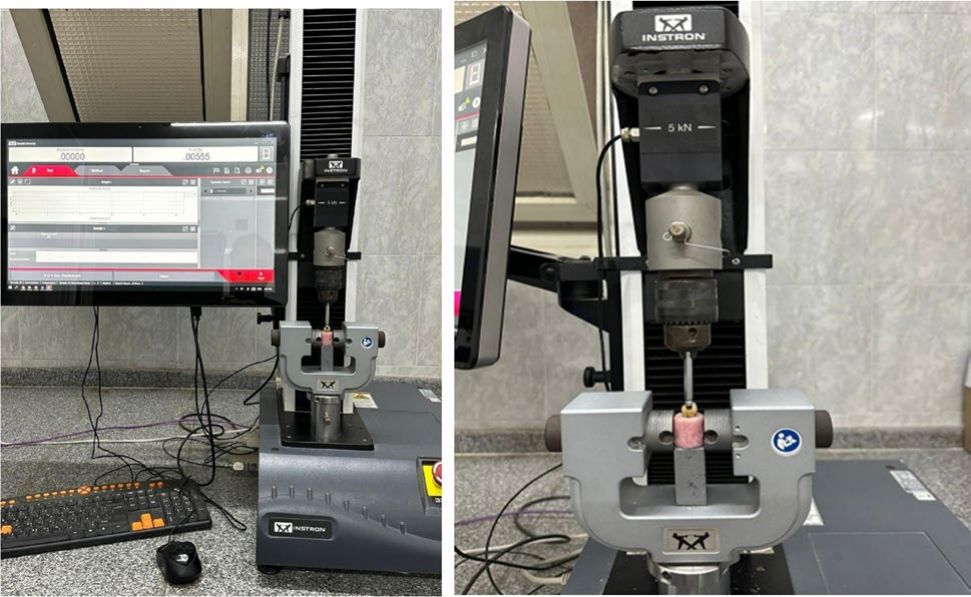
Testing set up

Fracture resistance was tested using a static compressive load-to-failure method in a universal testing machine (Instron, model 3345). A 5 mm diameter rounded steel indenter was applied vertically at the central fossa to contact both buccal and lingual cuspal inclines simultaneously at a crosshead speed of 1 mm/min until fracture occurred, and the peak load was recorded as the fracture resistance value in newtons. (Fig. 5) [15] Failure was detected by the first crack sound and confirmed by a quick drop in the load deflection curve recorded using computer software (Blue Hill Instron) [14]. 

Following failure, each tooth was carefully removed from the acrylic block without cutting the resin. The samples were immersed in warm water (~ 60 °C) to soften the wax layer used for periodontal ligament simulation, allowing gentle removal of the teeth without disturbing the fracture surfaces. [3] Then they were visually examined and photomicrographed via a digital camera (Nikon D850, Japan). Fracture patterns were classified into four types; Type I: Cusp or resin composite fracture above the CEJ; Type II: Vertical fracture at one or two cusps that did not extend into the root; Type III: Vertical fracture at one or two cusps below the CEJ extending into the root and type IV: Vertical longitudinal fracture dividing the crown into two pieces extending into the root or bifurcation. Types I and II were considered repairable modes of failure, whereas types III and IV were considered nonrepairable modes [14]. 

#### Statistical analysis

Data were analyzed using SPSS version 25.0 (IBM, USA). The normality of data distribution was verified using the Shapiro–Wilk test. One-way ANOVA was performed to compare fracture resistance among groups, followed by Tukey’s post-hoc test for pairwise comparisons. The significance level was set at *p* < 0.05 [20]. 

## Results

### Fracture resistance results

**Fig. 6:**
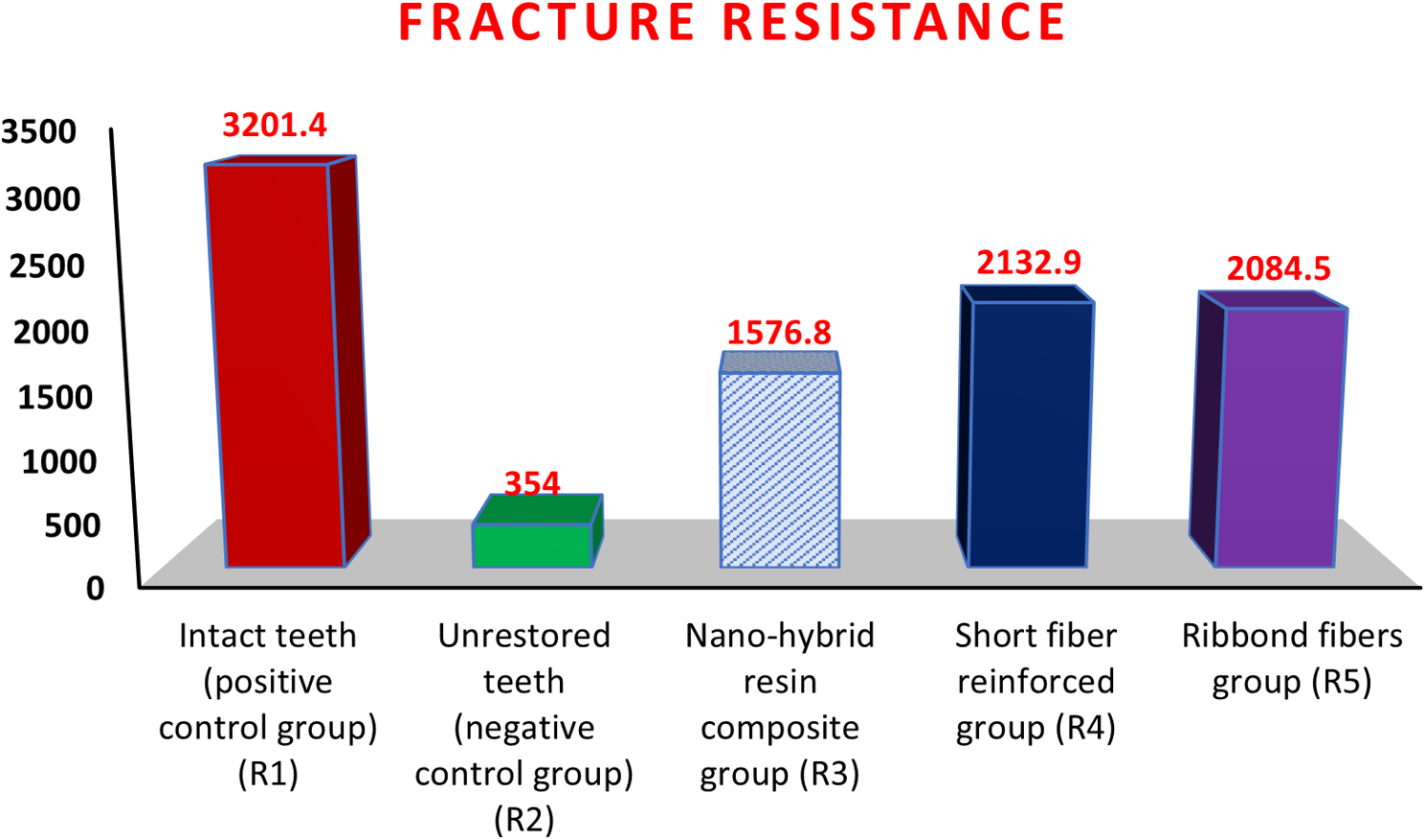
Bar chart representing the fracture resistance for different tested groups

**Fig. 7:**
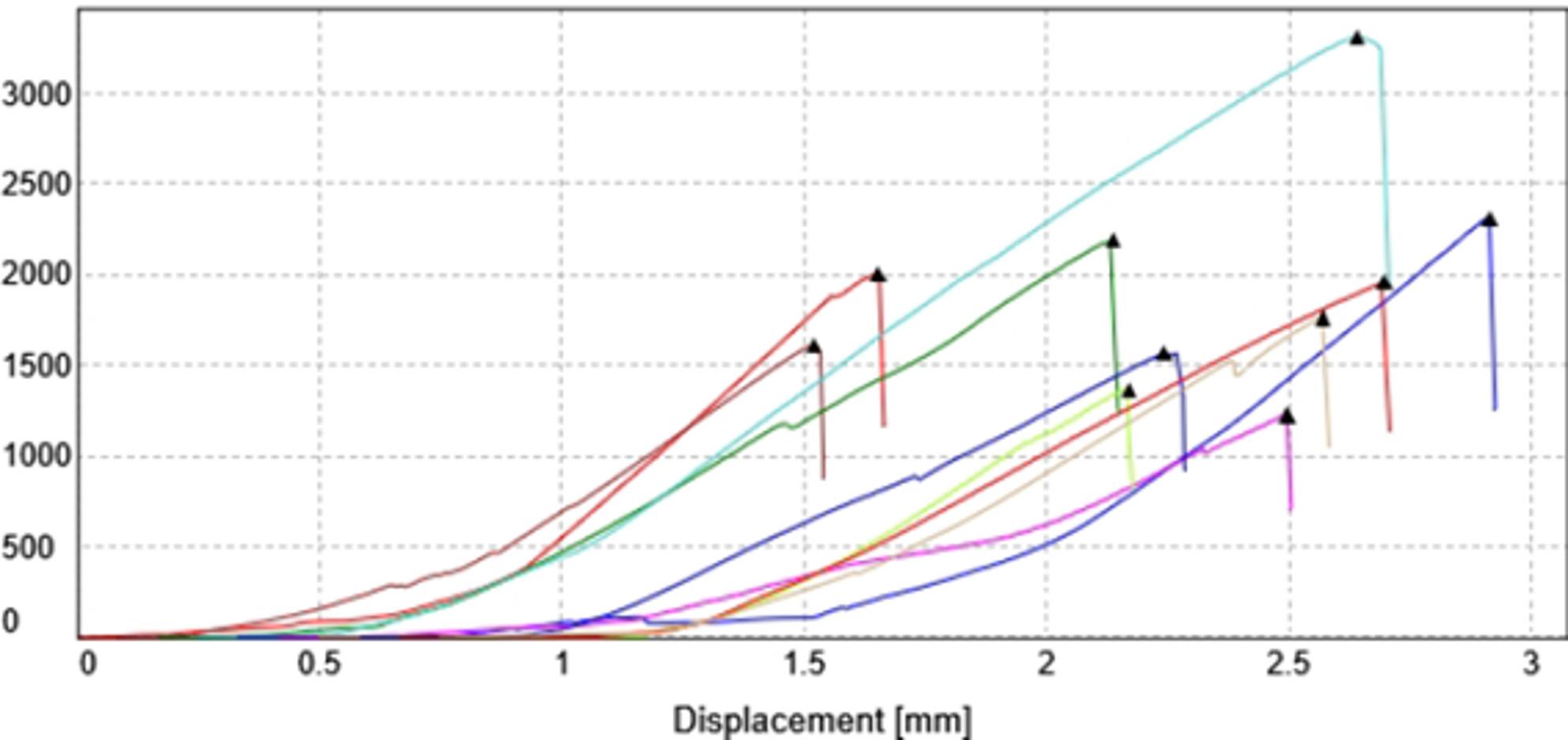
Load deflection curve

The results in Table 2, and Figs. 6 and 7 revealed that intact group (R1) showed the highest fracture resistance, followed by the SFRC (R4) and Ribbond (R5) groups, which did not differ significantly (*p* = 0.436). Both fiber-reinforced groups exhibited significantly higher values than the nanohybrid (R3) and unrestored (R2) groups (*p* < 0.001).

**Table 2:** Fracture resistance mean and standard deviation (SD) in different tested groups. Figs. 6 and 7

Groups	mean	SD	F test	*P* value
1.Intact teeth(positive control group) (R1)	3201.4^a^	123.4	944.708	<0.001**
2.Unrestored teeth (negative control group) (R2)	354.0^d^	32.8		
3.Nanohybrid resin composite group (R3)	1576.8^c^	118.3		
4.Short fibers reinforced group (R4)	2132.9^b^	169.5		>0.001
5.Ribbond fibers group (R5)	2084.5^b^	177.2		

Different superscript letters mean significant difference for pairwise comparison (each two groups) at *P*<0.05

^a^Indicating the highest mean value while^d^indicating the lowest mean value

**, means significant difference between groups at *P*<0.05

### Failure mode frequency results

Repairable failures (types I and II) were defined as fractures confined above the cementoenamel junction (CEJ) that allowed restoration repair or replacement, while non-repairable failures (types III and IV) extended below the CEJ or caused vertical root fracture, rendering the tooth non-restorable [14].

**Fig. 8:**
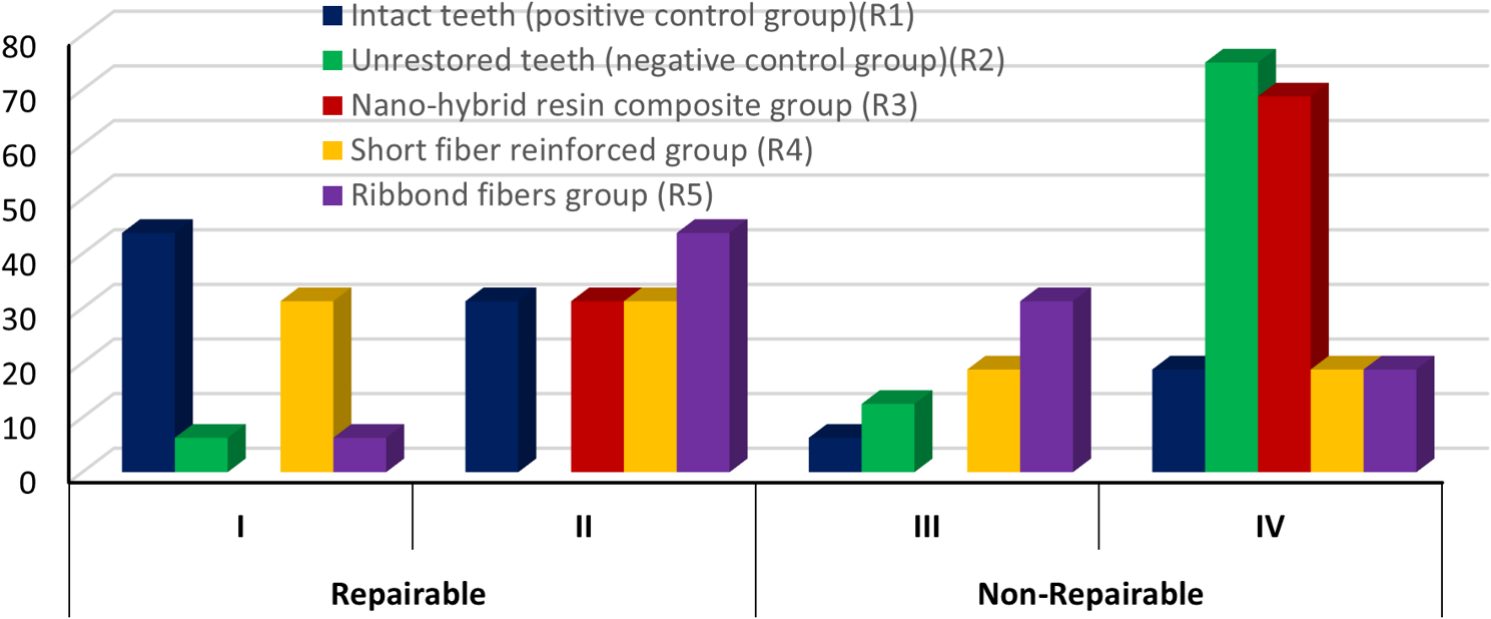
Bar chart representing failure types for different tested groups

**Fig. 9:**
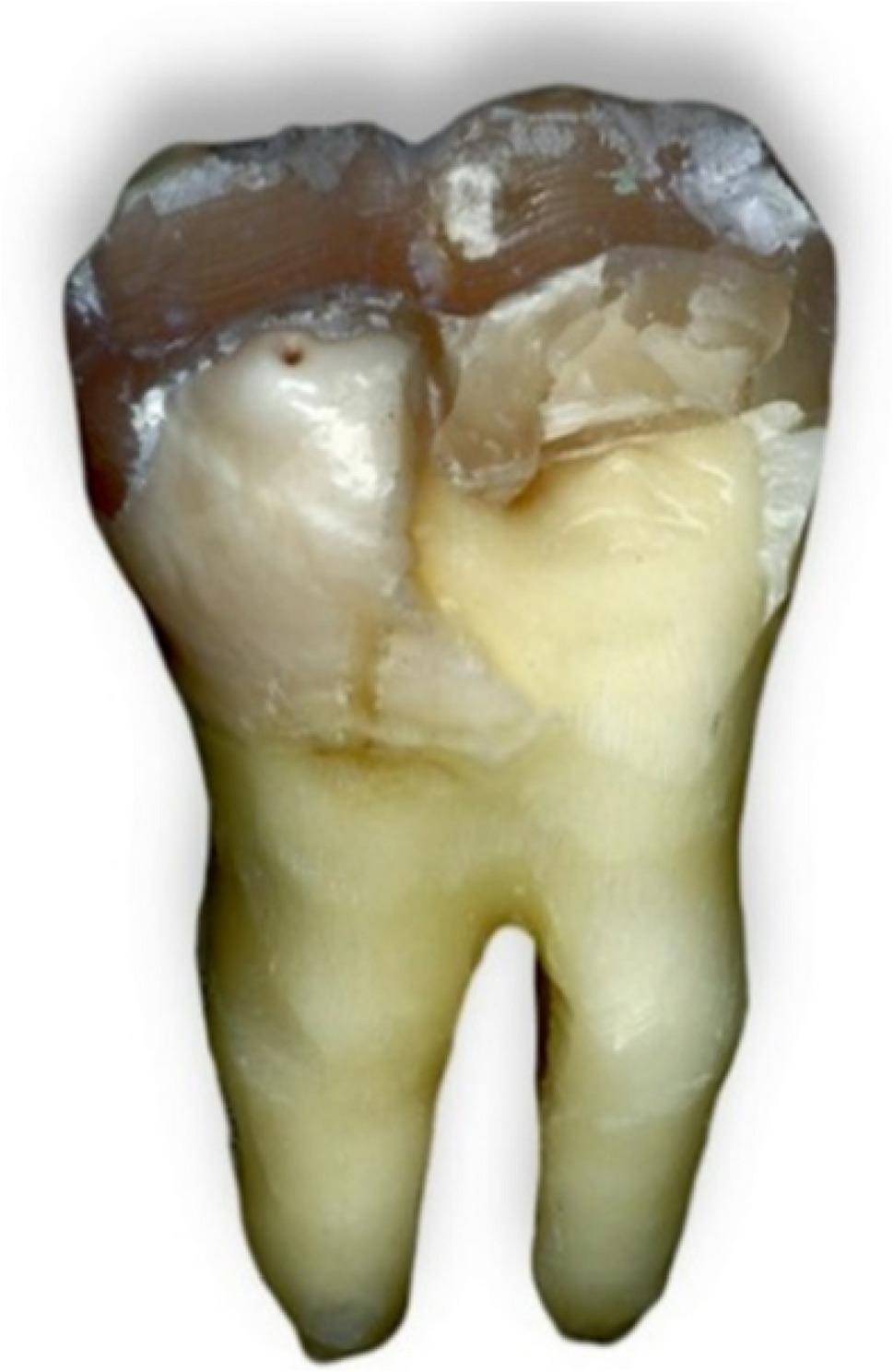
Failure pattern type I

**Fig. 10:**
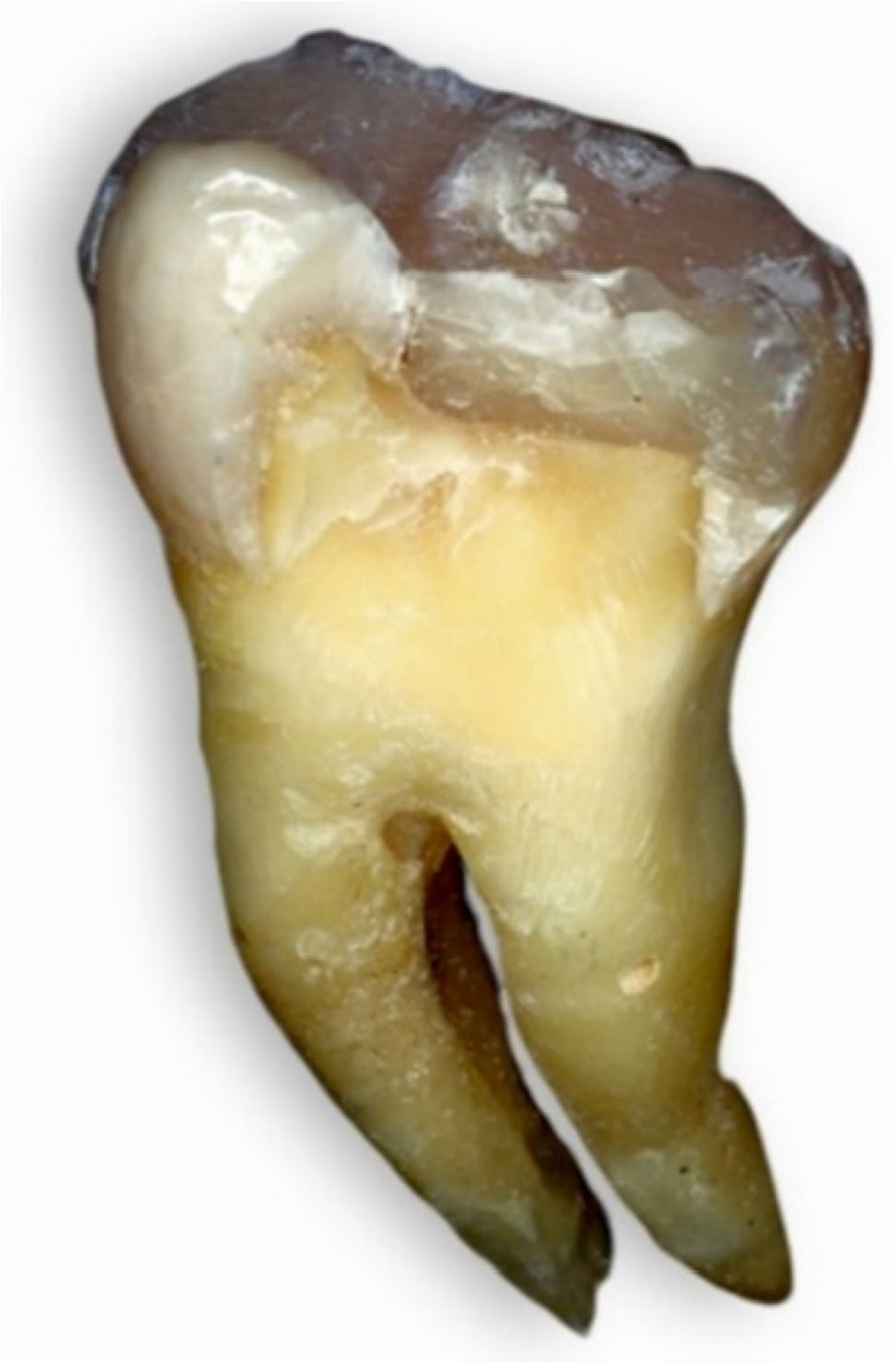
Failure pattern type II

**Fig. 11:**
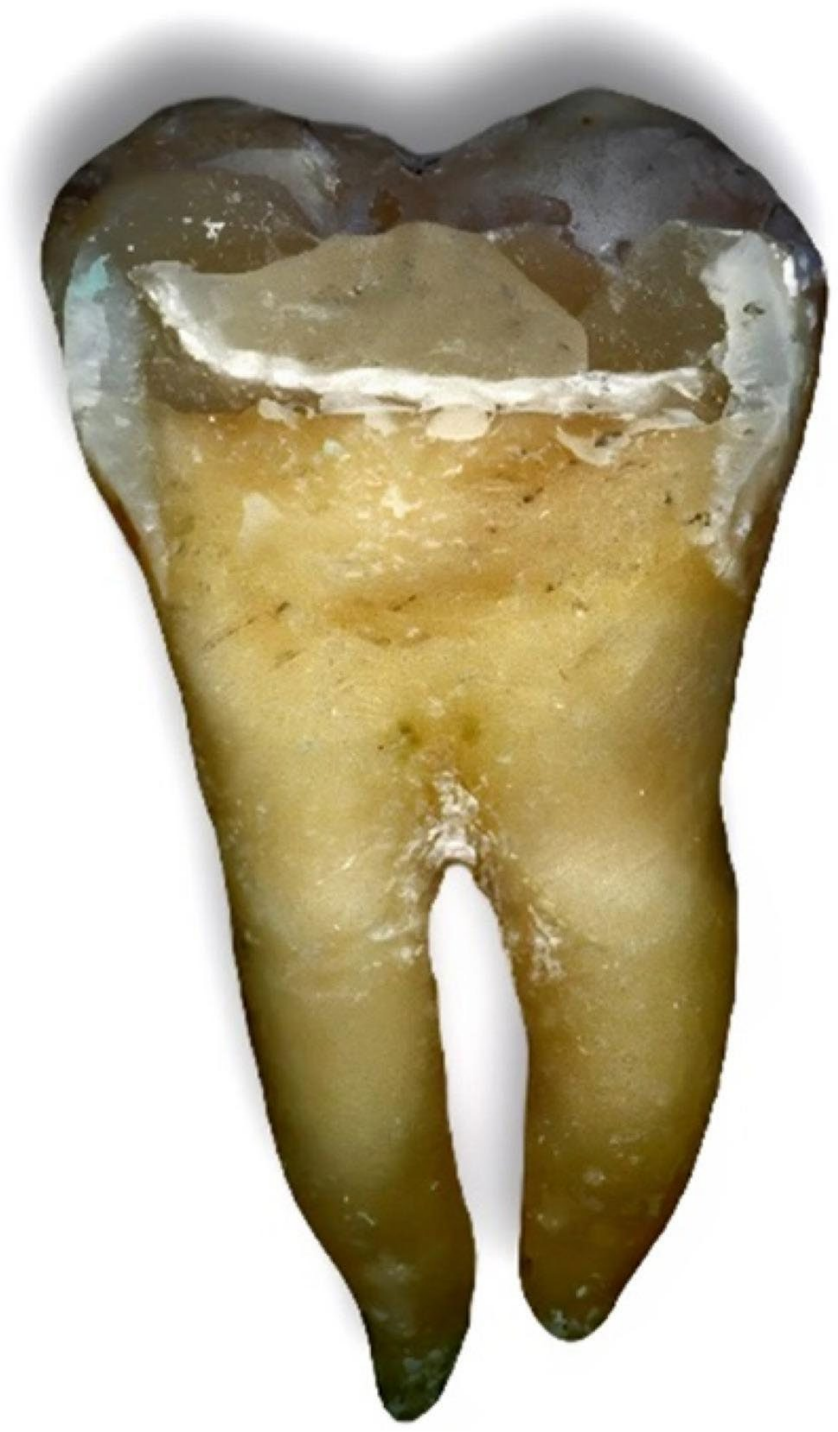
Failure pattern type III

**Fig. 12:**
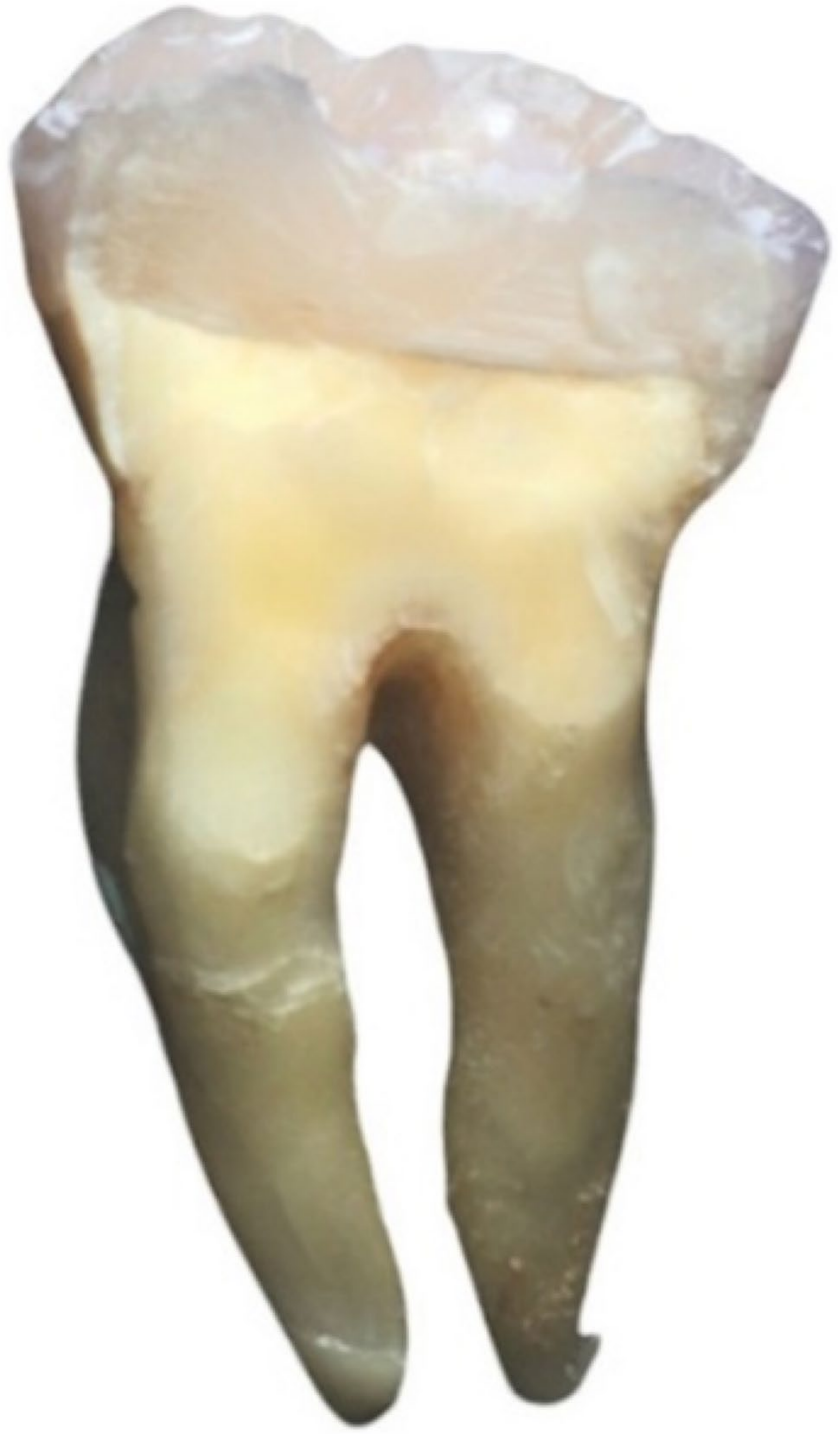
Failure pattern type IV

The results in Table 3 and Fig. 8 showed that intact teeth (R1) group showed predominantly repairable failure patterns, whereas the unrestored teeth (R2) exhibited mainly catastrophic (non-repairable) fractures. The nanohybrid composite group (R3) presented mostly non-repairable failures (type IV), whereas both the SFRC (R4) and Ribbond (R5) groups demonstrated a more favorable shift toward repairable failure types. Among all groups, the difference in the frequency of repairable failures (types I and II) was not statistically significant (*p* = 0.087), whereas non-repairable failures (types III and IV) showed a significant intergroup difference (*p* = 0.015). Figures 9, 10, 11 and 12.

**Table 3:** Failure type for different tested groups Figs. 7 and 8

	Repairable N (%)				Non-Repairable N (%)			
Groups	I	II	Total	P value	III	IV	Total	P value
Intact teeth (positive control group) (R1)	7(43.8)	5 (31.3)	12(75)	0.56	1(6.3)	3(18.8)	4(25.0)	0.317
Unrestored teeth (negative control group) (R2)	1 (6.3)	1(6.3)	2(12.5)	1.00	2(12.5)	12(75)	14(87.5)	0.0075*
Nanohybrid resin composite group (R3)	-	5 (31.3)	5(31.3)	0.025*	-	11(68.8)	11(68.8)	0.0009*
Short fibers group (R4)	5 (31.3)	5(31.3)	10(62.5)	1.00	3(18.8)	3(18.8)	6(37.5)	1.0
Ribbond fibers group (R5)	1(6.3)	7(43.8)	8(50)	0.033*	5(31.3)	3(18.8)	8(50)	0.479
Chi square	8.12487				12.32			
P value	0.08710				0.015*			

*, means significant difference between groups using Chi square at *P*<0.05

### Failure mode results

**Fig. 13:**
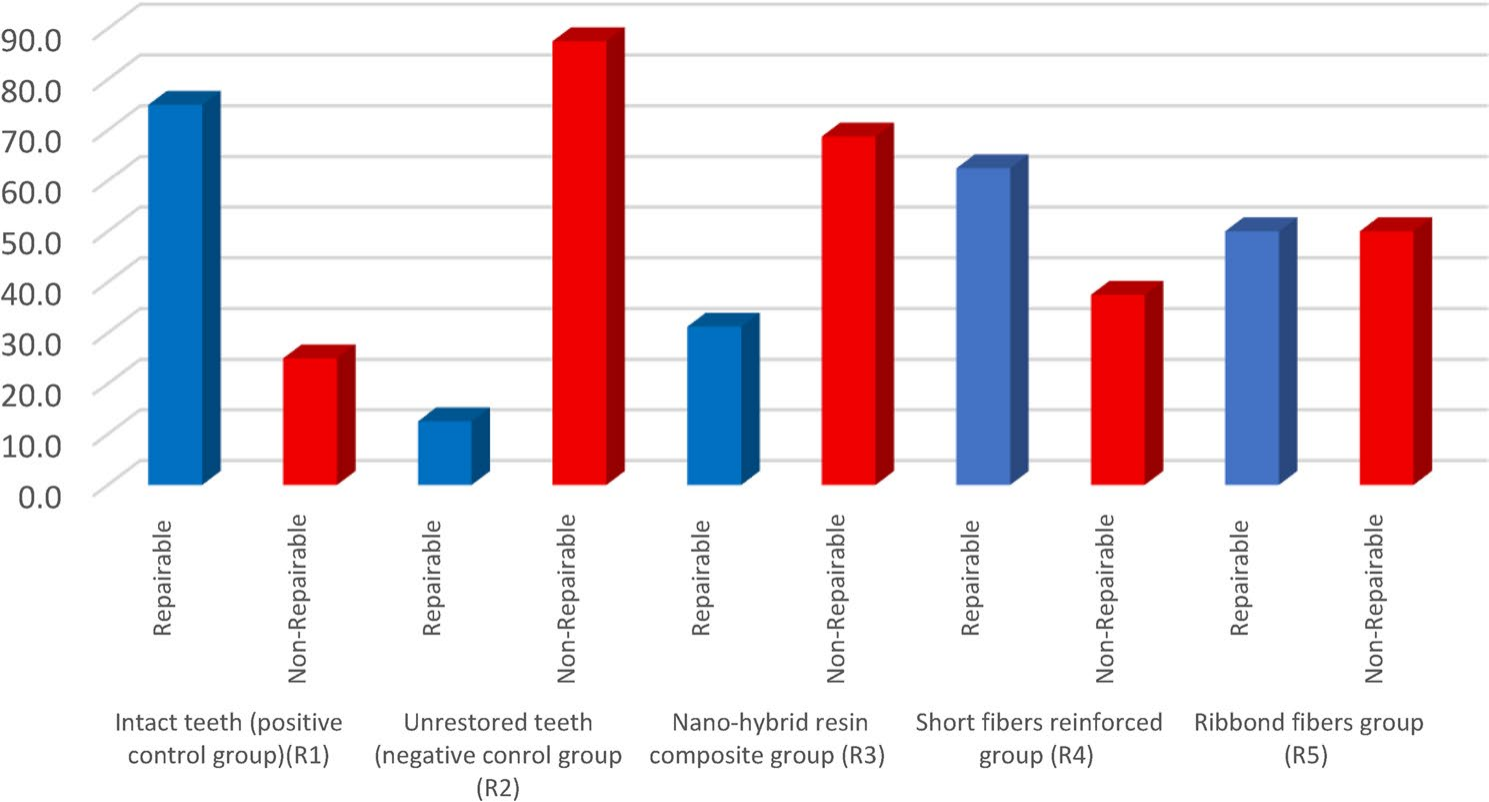
Bar chart representing failure mode for different tested groups

Chi-square analysis revealed a significant difference among the tested groups (*p* = 0.05). The results in Table 4 and Fig. 13 revealed that intact teeth group (R1) showed the highest proportion of repairable fractures (75%), followed by the SFRC (R4) and Ribbond (R5) groups, which also demonstrated a more favorable failure pattern compared to the nanohybrid composite (R3) and unrestored teeth (R2) groups. The negative control group (R2) exhibited the lowest repairable failure rate (12.5%). Within-group comparison showed significant differences between repairable and non-repairable failures only in R1 (*p* = 0.045) and R2 (*p* = 0.0027), while no significant differences were detected within R3, R4, or R5 (*p* > 0.05).

**Table 4:** Failure mode frequencies of different tested groups. Figures 8 and 13

Groups		N	%	Chi square	P values
Intact teeth (positive control group) (R1)	Repairable	12	75.0	4	0.045*
	Non-Repairable	4	25.0		
Unrestored teeth (negative control group) (R2)	Repairable	2	12.5	9	0.0027*
	Non-Repairable	14	87.5		
Nanohybrid resin composite group (R3)	Repairable	5	31.3	2.25	0.133
	Non-Repairable	11	68.8		
SFRC group(R4)	Repairable	10	62.5	1.00	0.317
	Non-Repairable	6	37.5		
Ribbond fibers group (R5)	Repairable	8	50.0	0.285	0.593
	Non-Repairable	8	50.0		
Chi square		15.88			
P value		0.00317**			

**, means significant difference between groups using Chi square at *P*<0.05

^*^,means significant difference intragroup

## Discussion

One of the dilemmas in restoring posterior teeth with wide MOD cavities is the fracture of cusps, which contributes to the compromised mechanical properties of restored teeth. This occurs due to microcrack propagation caused by repeated occlusal loading, which gradually separates the cusps. Consequently, reinforcing these teeth is important for increasing their fracture resistance [21, 22]. During daily dental practice, restoring these teeth is usually performed through either direct or indirect restorations.

It worth noting that direct restoration via resin composites has the advantages of preserving more natural tooth structure and increasing the opportunity for repair [23]. However, occlusal wear, secondary caries and fracture are common reasons for the failure and replacement of such restorations [24, 25] On the other hand, indirect restorations offer superior contouring of proximal surfaces, occlusal contacts, increased wear resistance, and decreased polymerization shrinkage. However, they come with drawbacks such as higher costs, the need for two appointments, need for temporary restoration, and limited repair potential [26]. Therefore, several reinforcement techniques have been introduced over the past decades, including glass fibers, fiber substructures, whiskers, and ceramic fillers [27, 28]. Fiber reinforcement has been introduced to improve resin composite restorations in restorative dentistry by strengthening them internally and reducing the degree of fractures [6]. Multiple types of fibers are utilized for such purposes, such as polyethylene ribbon and glass fiber-reinforced composites, which are the most commonly utilized types. Both types are crucial in enhancing the fracture resistance of mutilated teeth [29] as well as in improving the microleakage and marginal integrity of the restorations [30, 31, 32]. 

In the present study molar teeth with wide and deep MOD cavities were prepared for all groups to simulate the clinical scenario except for the positive control group which was left intact. A SFRC (everX Flow) was used in the current study as the manufacturer shown to have high mechanical properties regardless of its low viscosity [33]. It was placed in 2 mm increments and covered by a nanohybrid resin composite, as recommended by the manufacturer. The manufacturer showed that everX Flow is a strong base material in areas of high stress to provide support for large restorations and replicate the stress-absorbing properties of dentin [8]. Although SFRCs have been reported to perform adequately even without a surface coverage in proximal regions, in the present study they were applied as a base material and covered with a nanohybrid composite layer to ensure standardized surface finishing and comparable mechanical properties [11]. On the other hand, Ribbond Ultra, which is an ultra-high molecular weight polyethylene (UHMWP) fiber, was the second type of fiber reinforcement used in the study. It is treated with cold gas plasma during manufacturing to improve its chemical bonding with restorative materials [34, 35]. It was placed against the cavity floor to cover the entire pulpal floor and extend up to the mesial, and distal cavity walls in a U-shaped pattern as the manufacturer recommended and claimed that Ribbond’s success was attributed to its patented leno weave, which sets it apart from other fiber reinforcements.

Thermocycling was performed to reproduce thermal fluctuations occurring intraorally during daily temperature changes. This protocol simulates short-term thermal fatigue and interfacial stress at the tooth–restoration interface. Although it provides standardized preloading aging, it does not fully replicate the complex oral environment, which involves cyclic masticatory forces, salivary enzymes, and long-term moisture effects. Therefore, the findings should be interpreted within the limits of short-term thermal aging. Thermocycling was limited to 1000 cycles, representing a short-term aging protocol equivalent to approximately one month of intraoral service [36]. This duration was sufficient to assess the initial mechanical response of the restorations, while long-term aging and fatigue behavior should be addressed in future studies [37, 38]. 

Then, evaluation of the fracture resistance in the current study was carried out for all the tested groups. It goes without saying that the combination of cyclic loading and inherent flaws in the restorative system can cause cumulative damage, potentially leading to early failure. Therefore, this assessment method reflects the restorative material’s resistance to intraoral compressive and tensile forces that are generated during function. Consequentially, the restorative material must have high compressive strength to withstand these forces. Therefore, researchers commonly use compressive strength tests for estimating the efficacy of restorative materials in oral environments [38]. 

The results of the present study revealed that the highest mean value of fracture load was recorded in intact teeth (positive control group), (R1) followed by the SFRC group (R4), followed by Ribbond fibers group (R5) then the nano-hybrid resin composite group (R3), respectively, while, the lowest mean value was found in the unrestored teeth (negative control group), (R2) with a statistically significant difference between all of them. The highest mean value of fracture load of the intact teeth (positive control group, R1) could be related to the presence of an intact occlusal table, along with intact marginal ridges and oblique ridges, which creates a continuous circle of tooth structure that strengthens and preserves the tooth’s integrity [39]. Meanwhile unrestored teeth (negative control group, R2) recorded the lowest mean value of fracture load as cuspal separation and loss of the marginal ridge have led to loss of teeth integrity and rigidity [40]. 

Additionally, it is worthy to mention that short fibers reinforced group (R4) and Ribbond fibers group (R5) revealed significantly higher mean values than that recorded by the nano-hybrid resin composite group (R3). These inferior results of the nanohybrid resin composite group could be related to complaining of the resin composite material of low toughness values. Although compressive forces act on all restorations, stress distribution differs by material type. In conventional composites, stresses concentrate along cavity walls and cuspal margins, promoting crack initiation. In contrast, fiber-reinforced restorations can dissipate these stresses more efficiently through the fiber network, limiting crack propagation and improving fracture resistance [41]. 

Evaluation of the results of the Ribbond fibers group (R5) revealed a significantly greater compressive strength than did the nanohybrid resin composite group. This result could be related to the lock-stitch features of the Ribbond fibers that effectively distribute forces throughout their characteristic weave without transferring the stresses back into the resin. Ribbond’s weaves provide excellent handling and almost no memory, allowing close adaptation to tooth contours. This results in a thinner adhesive interface and improved shear bond strength. By reducing the resin layer thickness between fibers and tooth, it also minimizes stress concentration and C-factor effects [18]. Furthermore, Polyethylene fibers in Ribbond have dual functions as they act as a stress-absorbing layer to redirect cracks and fractures [42], at the same time they internally splint the tooth to increase fracture resistance [43]. More specifically, Ribbond Ultra which was utilized in the present study is the thinnest of all currently available Ribbond fiber reinforcement with 0.12 mm thickness, which enables it to boast the highest flexural modulus and peak load [18]. 

On the other hand, the results of the present study showed that the SFRC group (R4) had slightly higher fracture resistance than the Ribbond fibers group (R5). These findings indicate that the utilized everX Flow in the R4 group was manufactured using Optimal Aspect Ratio technology, which uses shorter and thinner fibers that significantly improve manipulation while still reinforcing the material effectively. According to the literature, the mechanical properties of short fibers reinforced resin composites are affected by parameters like aspect ratio, critical fiber length, fiber loading, and orientation [44]. Aspect ratio (length/diameter ratio) by its turn influences the tensile strength, flexural modulus, and reinforcement effectiveness. Also, E-glass microfibers in everx Flow have critical fiber lengths to diameter ratios [140 μm in length and 6 μm in diameter] [45]. As documented, effective stress transmission from the matrix to the fibers in fiber reinforced resin composites, necessitates that the fiber length should meet or exceed the critical fiber length and adhere to the specified aspect ratio [45]. 

Additionally, an essential factor in enhancing the fracture resistance of short fibers reinforced resin composite materials is the effective coating of the fibers and filler content with a silane coupling agent, in contrast to Ribbond fibers, which are treated with cold gas plasma only. This silanization of the short fibers reinforced resin composite material plays a crucial role in maintaining the bond between its filler content and the matrix in the case of the everX Flow group. Additionally, this procedure controls the surface energy of the fibers so sufficient adhesion between glass fiber and matrix resulted in good load transfer between them, which ensures that the load will transfer to the stronger fibers [46]. This phenomenon appeared to improve the everX Flow material’s resistance to crack propagation by enhancing the individual fiber’s ability to act as a crack stopper.[3] Also, the innovative full-coverage Silane Coating technology of the fibers has significantly improved the silanization process for both particle fillers and fibers, allowing for increasing the filler content without increasing the viscosity of the material. Leveraging these advancements, everX Flow has high compressive strength and a favorable mode of failure.

Additionally, the current study evaluated the distribution of fracture modes to assess the restoration status, which is crucial for determining whether repair, replacement or tooth extraction is necessary. Recording of repairable failure by the restorative system is of paramount importance as it considered as a conservative approach to improve the durability of dental restorations compared to complete replacement or tooth extraction, as noted by dental practitioners [47]. The results of the present study showed that intact teeth (positive control group, R1) showed the highest significant frequency of reparability (75%), meanwhile unrestored teeth (negative control group, R2) revealed the lowest frequency of repairability (12.5%). These findings of intact teeth can be clarified due to regular distribution of load applied to the tooth along the intact dental structures as mentioned before. Meanwhile, exerting a load on unrestored teeth can create a wedge effect between the buccal and lingual cusps, leading to severe unrepairable fractures. Also, larger cavity dimensions can result in greater cuspal deflection, where the cavity floor acts as a fulcrum for cusp bending, with the length of the cantilever cusp wall increasing as the cavity depth increases. [3]

Furthermore, evaluation of the failure mode results of the short fibers reinforced group (R4) recorded 62.5% reparability, followed by the Ribbond fibers group (R5) with 50%, while the nanohybrid resin composite group (R3) recorded 31.3%. The high frequency of repairable failures recorded in SFRC group could be related to that the fibers hinder crack growth by creating interlocking bridges that dissipate energy through fiber pullout, leading to a gradual failure rather than sudden breakage. Also, the random orientation of the fibers in the short fibers reinforced resin matrix in the forms a network that enhances the material’s resistance to crack propagation and reduces stress intensity at the crack tip, preventing unstable crack propagation [48]. The use of fiber-reinforced composites improved reinforcement efficiency by primarily preventing fractures in the enamel. This may be due to its ability to halt crack propagation in the cervical direction and redistribute 3-dimensional stress without causing debonding or fractures in the cavity or restoration [49]. 

Additionally, the fibers in SFRC group can redirect and halt the progression of fractures within the composite due to stress transfer from the weak matrix to the strong fibers, depending on the fiber’s length and diameter [49]. Based on all previously mentioned mechanisms, a better mode of failure could be expected in such samples restored with such material.

Therefore, based on all previously mentioned results the null hypothesis of the current study was rejected. However, static load-to-failure testing was only performed to assess the initial fracture resistance of the restorations. Although this method provides standardized and reproducible results, it does not account for cyclic fatigue and long-term degradation occurring in the oral cavity. Thermocycling protocol was limited to 1000 cycles between 5 °C and 55 °C to simulate short-term thermal aging equivalent to approximately one month of intraoral function. This protocol allowed a controlled evaluation of early mechanical behavior but did not fully replicate extended clinical aging.

A negative control group (unrestored teeth) was included to establish a baseline reference for the maximum loss of structural integrity after cavity preparation and to highlight the reinforcing effect of different restorative approaches. The study did not include a group restored with millimeter-scale SFRC (everX Posterior), which could have provided additional comparison regarding the influence of fiber length on reinforcement efficiency. This aspect should be explored in future studies involving different fiber architectures and long-term fatigue protocols. Therefore, further studies are required to evaluate the long-term performance of the tested materials under more clinically relevant conditions, including the effects of thermal cycling and dynamic loading, aging in oral fluids and chewing simulation are used to more accurately simulate the complicated oral environment.

### Clinical implications

Clinically, both short fiber-reinforced and polyethylene fiber-reinforced composites can enhance the fracture resistance of extensive MOD restorations and shift failure patterns toward more repairable fractures. This suggests that using internal fiber reinforcement may help preserve tooth structure and facilitate repair rather than full replacement after failure.

The choice between SFRC and Ribbond reinforcement should be guided by cavity morphology and clinical accessibility: SFRC may be more suitable for internal reinforcement in wide cavities, whereas Ribbond fibers can provide cusp-splinting benefits in cases of thin remaining walls. However, these in-vitro findings should be confirmed by long-term clinical studies before routine clinical recommendation.

From a clinical handling perspective, SFRC offers easier application and adaptation within the cavity compared to Ribbond fibers. SFRC can be placed and light-cured in increments similar to conventional composites, ensuring better adaptation to cavity walls and fewer voids. In contrast, Ribbond fibers require pre-cutting, wetting with resin, and careful placement to prevent displacement or folding, which may complicate adaptation in deep or narrow cavities. However, Ribbond provides excellent control of fracture propagation when properly positioned across the cavity floor or cuspal area. Therefore, the choice between SFRC and Ribbond may depend on operator preference, cavity morphology, and ease of clinical manipulation.

### Limitations and Future Directions

This in vitro study provides valuable insights but is inherently limited by the absence of dynamic oral conditions such as mechanical wear from mastication. Future studies should incorporate multifactorial aging models, including brushing abrasion, chewing simulation and pH cycling, to better simulate the oral environment. Additionally, evaluating other types of reinforcements could provide comparative data for more informed material selection in clinical practice.

## Conclusions

Short glass and polyethylene fiber-reinforced composites have a positive impact on the fracture resistance and repairability of restored MOD cavities.

## Data Availability

The datasets used and/or analyzed during the current study are available from the corresponding author upon reasonable request.
